# Detection of Cervical Intraepithelial Neoplasia Using Hyperspectral Tissue Signatures

**DOI:** 10.1109/JTEHM.2025.3630878

**Published:** 2025-11-10

**Authors:** Ovidiu Jurjuţ, Martin Weiss, Yannick Daniel, Sabine Matovina, Felix Neis, Katharina Rall, Katharina Schöpp, Melanie Henes, Walter Linzenbold, Sara Y. Brucker, Jürgen Andress

**Affiliations:** Department of Women’s HealthUniversity Hospital Tübingen Tübingen 72076 Germany; NMI Scientific Medical InstituteEberhard Karls University of Tübingen Reutlingen 72770 Germany; Erbe Elektromedizin GmbH Tübingen 72072 Germany

**Keywords:** Colposcopy, biomarkers, spectroscopy, LLETZ, interobserver variability, particularly in settings with limited access to expert colposcopists

## Abstract

Cervical intraepithelial neoplasia (CIN) represents a spectrum of premalignant lesions requiring accurate early detection to prevent progression to invasive cervical cancer. Colposcopy with visual inspection using acetic acid (VIA) is the gold standard for CIN assessment but suffers from substantial interobserver variability, limiting diagnostic consistency. We evaluated hyperspectral imaging (HSI) as an objective, non-invasive method for characterizing CIN-related tissue changes. This prospective proof-of-principle clinical study enrolled women with histologically confirmed CIN3 indicated for large-loop excision of the transformation zone (LLETZ). Standardized colposcopic images following VIA were obtained and annotated independently by five certified colposcopists according to IFCPC Rio 2011 classification. These annotations served as pathological tissue region references and were quantitatively assessed using intersection over union metrics to evaluate interobserver agreement. HSI was performed immediately prior to LLETZ using the TIVITA Tissue System, capturing spectral reflectance data across 500–995 nm in 100 wavelength bands. Spatial correspondence between colposcopic and hyperspectral images was achieved through homography transformation based on landmark alignment, allowing expert annotations to be projected into the HSI domain. Reflectance spectra from annotated areas were averaged to calculate four proprietary HSI-derived tissue indices, which revealed significantly higher values in CIN-affected regions compared to healthy tissue (p <0.01, Wilcoxon signed-rank test), suggesting increased vascularization and water content. Our findings highlight conventional colposcopy limitations due to examiner subjectivity and support HSI’s potential to provide reproducible, quantitative biomarkers for CIN. HSI integration into clinical workflows may enhance cervical cancer screening objectivity and enable reliable diagnostics in resource-limited settings. Clinical and Translational Impact Statement— Hyperspectral imaging enables objective detection of cervical intraepithelial neoplasia and could improve diagnostic accuracy while reducing unnecessary biopsies

## I. Introduction

Cervical Intraepithelial Neoplasia (CIN) encompasses a spectrum of premalignant epithelial lesions that can progress to cervical cancer if not detected and treated early. Organized screening programs typically incorporate high-risk human papillomavirus testing and cytological evaluation by Pap smear, with abnormal findings requiring colposcopic assessment and histopathological confirmation [1]. These have contributed to a substantial decline in cervical cancer incidence, yet the disease remains the fourth most prevalent cancer among women worldwide, accounting for approximately 270,000 deaths annually [2].

Colposcopy, including visual inspection with acetic acid (VIA), plays a pivotal role in CIN diagnosis and management. However, its accuracy is limited by strong examiner dependence, with reported false-negative rates ranging from 13% to 69% even among trained specialists [3], [4]. Variability in examiner experience, lesion appearance, and subjective pattern recognition all contribute to diagnostic accuracy. Additionally, access to colposcopy services remains unevenly distributed, with specialized facilities often concentrated in tertiary healthcare centers. This substantial diagnostic variability underscores the urgent need for standardized, objective methods to support reliable CIN assessment, particularly in settings where specialized expertise is lacking.

HSI has emerged as a promising modality to improve diagnostic precision in a variety of clinical contexts, including tissue differentiation, assessment of blood perfusion, wound healing, and inflammatory processes [5], [6], [7], [8], [9]. By capturing the reflectance of light across a wide spectral range, HSI enables non-invasive, marker-independent tissue characterization, potentially providing molecular and structural information beyond the capabilities of conventional colposcopy. While its potential has been demonstrated in several fields, its application to cervical pathology remains limited [10].

The objective of this proof-of-concept clinical study is to assess whether HSI is suitable for distinguishing CIN-affected from healthy cervical tissue. The results show significant spectral differences between CIN-affected and healthy tissue and confirm the potential of HSI for reducing interobserver variability in colposcopic evaluation. The development and clinical validation of such methods could facilitate more standardized CIN detection, enabling less experienced healthcare providers to conduct reliable screenings and ultimately improving access to early cervical cancer prevention strategies worldwide.

## II. Methods and Procedures

### A. Patient Inclusion Criteria

This study was designed in accordance with the Declaration of Helsinki, approved by the local ethics committee at the Medical Faculty of the University of Tübingen (650/2017BO1) and registered at the German Clinical Trials Register (DRKS.de, DRKS-ID: DRKS00013478). Patients with histopathologically confirmed CIN3 and clinical indication for large-loop electric excision of the transformation zone (LLETZ), female gender, and minimum age of 18 years were included. Included patients were informed about the procedures in detail and provided their written informed consent.

### B. Clinical and Study Workflow

Patients with abnormal Pap smear results were referred for colposcopic evaluation. At the first visit, participants underwent colposcopic examination with VIA, during which the cervix was imaged with a 1080p camera and biopsies were obtained. Biopsy specimens were histopathologically assessed to confirm the presence of CIN3. At the second visit, patients with confirmed CIN3 provided informed consent for study inclusion and were scheduled for LLETZ. The intervention was performed under general anesthesia and included HSI imaging of the cervix, VIA, and LLETZ excision. Following data acquisition, areas with tissue transformation were annotated in the colposcope images by medical examiners. Landmarks were then defined in both colposcope and HSI images to enable their alignment and the transfer of annotations into the HSI space. Finally, tissue property values derived from HSI were compared between CIN-affected and healthy regions.

### C. Annotation of CIN in Colposcopy-Derived Images

Images of the cervix were taken using a conventional colposcopic working station (3MV, Leisegang, Germany) after 5% Acetic Acid application. The objective of the camera was placed at approximately 30 cm from the patient such that the cervix was properly in focus. High resolution images (full HD, IMAGE1 S^TM^, Karl Storz, Germany) of the cervix were taken under halogen lighting from the colposcope.

Images were then retrospectively inspected by up to five expert medical examiners (all certified by the Working Group of Cervical Pathology and Colposcopy, AG-CPC, within the German Society of Gynecology and Obstetrics, DGGG). The examiners were tasked with annotating specific tissue areas corresponding to minor and major changes of the cervix according to VIA, and with reporting according to the Colposcopy Terminology of the International Federation for Cervical Pathology and Colposcopy (IFCPC) [11]. Areas with major and minor tissue changes were marked separately. Additionally, an area without visible tissue changes was marked for control purposes. The examiners performed the annotations independently, in order to minimize bias.

To visualize the consensus among all examiners for one patient, a 2D histogram of their annotations was normalized to the number of examiners. The resulting values ranged between 0 and 100%, with 0% corresponding to areas that were not marked by any examiners and 100% corresponding to areas marked by all examiners. This was done separately for areas with major and minor tissue changes, as well as for the entire area with changed tissue, irrespective of the degree of tissue change.

To quantify consensus among pairs of examiners for one patient, the intersection over union (IoU) metric was used. The IoU, also known as the Jaccard index, is the most commonly used evaluation metric in object detection benchmarks, such as PASCAL VOC [12] and MS COCO [13]. It quantifies the difference between the intersection and the union of two areas, yielding a score between 0, which corresponds to no overlap, and 1, which corresponds to a perfect overlap of the two areas. IoU values were averaged across patients to estimate overall consensus among examiners and detect any bias between examiners pairs, i.e. high consensus among specific examiners.

### D. Hyperspectral Imaging of the Cervix

Hyperspectral images were acquired using the TIVITA ® Tissue System (Diaspective Vision, Germany) [14] in a darkened room to minimize external light contamination. Homogeneous tissue illumination was provided by the six halogen lamps integrated around the system’s camera. The camera captured reflected light in the range of 500–1000 nm in 5 nm steps, with a resolution of $640\times 480$ pixels and a spatial resolution of approximately 0.45 mm/pixel. The system was calibrated using a color-checking palette at the start of each measurement day. Images were recorded at a distance of $50~\pm ~5$ cm from the tissue surface of patients in the lithotomy position. To enhance visibility of the CIN lesions, the cervix was manually externalized by lateral fixation using sutures after disinfection. HSI measurements were taken on the native state cervix, followed by VIA, colposcopy imaging and LLETZ under colposcopic guidance [10]. Due to the properties of the imaging system, the cervix occupied a small part of the imaged field of view. Only the HSI image area corresponding to the cervix was used for further analyses.

### E. Matching Colposcope and Hyperspectral Images

To analyse the cervix tissue from a hyperspectral perspective, tissue annotations from the colposcope images were mapped onto the HSI space. This process required establishing a correspondence between the colposcope image and the region of the HSI image containing the cervix. First, a medical expert identified and marked at least four landmarks in each pair of images. These landmarks were defined by tissue boundaries, pigmentation or morphology, or any other visual cue that could be recognized in both images. Next, the landmark coordinates were used as input to the findHomography function from the OpenCV Python library [15]. This function computes the perspective transformation matrix H that maps points from a source image (colposcope) to a destination image (HSI), defined by the relation:

\begin{align*} \left [{{\begin{array}{c} x_{i}^{\prime } \\ y_{i}^{\prime } \\ 1 \end{array}}}\right ] \sim H\left [{{\begin{array}{c} x_{i} \\ y_{i} \\ 1 \end{array}}}\right ]\end{align*}

where ( $x_{i}$, $y_{i}$) and ( $x'_{i}$, $y'_{i}$) represent the landmark coordinates in the source and destination images, respectively. The coefficients of the H matrix ( $h_{mn}$) were chosen to minimize the back-projection error:

\begin{align*} & \sum _{i}\left ({{x_{i}^{\prime }-\frac {h_{11} x_{i}+h_{12} y_{i}+h_{13}}{h_{31} x_{i}+h_{32} y_{i}+h_{33}}}}\right)^{2} \\ & \quad +\left ({{y_{i}^{\prime }-\frac {h_{21} x_{i}+h_{22} y_{i}+h_{23}}{h_{31} x_{i}+h_{32} y_{i}+h_{33}}}}\right)^{2}\end{align*}

The mapping was successful for all n= 11 image pairs. Using the H matrix, the tissue annotations from the colposcope images were mapped to HSI space coordinates. This transformation allowed the annotations to be superimposed onto the HSI image of the cervix, enabling the investigation of the spectral signatures underlying the annotated areas.

### F. Analysis of Hyperspectral Data

To reduce variability in the analysis caused by differences between examiners when annotating cervix tissue, a threshold was applied to keep only regions where examiner consensus exceeded 50%, i.e. areas were included only if at least half of the examiners agreed that the annotated tissue exhibited minor or major changes. Thresholding was applied exclusively to the CIN annotations. In contrast, regions annotated as healthy tissue were included in their entirety, regardless of examiner consensus. This approach was chosen because examiners were not instructed to mark all healthy tissue areas, but rather to annotate only a representative sample area.

Using the formulas provided by the manufacturer of the TIVITA® Tissue System, the proprietary tissue indices—Tissue Water Index (TWI), Tissue Hemoglobin Index (THI), Oxygen Saturation (StO2), and Near-Infrared Perfusion (NIR)—were computed for each pixel in the HSI image.

Next, the indices corresponding to the pixels within the thresholded, annotated areas were averaged to produce a representative index for each tissue type. Finally, the indices corresponding to CIN-affected and healthy tissue were compared across the entire patient population.

### G. Statistical Methods

To assess statistical differences between CIN-affected and healthy tissue, the Wilcoxon signed-rank [16] test was applied for each tissue index (*wilcoxon* function, Scipy 1.14.1). This non-parametric test tests whether two related paired measurements come from the same distribution. The Hodges-Lehmann estimator [17] of the median paired difference was calculated to provide a robust estimate of the typical difference between conditions. 95% confidence intervals (CI) for the Hodges–Lehmann estimate were obtained using nonparametric bootstrap resampling with 10000 repeats [18]. In addition, a standardized effect size based on the test statistic was reported to allow interpretation and comparison across studies [19]. Together, these analyses were used to provide both statistical significance and practical effect estimates for CIN-affected and healthy tissue.

### H. Data and Code Availability

The data used in this study are available upon reasonable request from the corresponding author. All analyses were performed using custom Python scripts, with support from widely used open-source libraries such as OpenCV, SciPy, NumPy, and Pandas. The code is also available upon request from the corresponding author.

## III. Results

### A. Delimitation of CIN Areas is Observer Dependent

To quantitatively assess the accuracy of identifying and demarcating cervical tissue affected by CIN, five certified medical experts were instructed to annotate specific tissue regions. These annotations corresponded to minor and major cervical changes based on VIA and were reported in accordance with the Colposcopy Terminology of the International Federation for Cervical Pathology and Colposcopy (IFCPC) Rio 2011 guidelines [11]. Additionally, for control purposes, each examiner identified a region of cervical tissue that exhibited no dysplastic changes. While the annotation of CIN-affected areas was comprehensive, the size and location of the control area were left to the examiner’s discretion. Examiners performed annotations independently across all patient images.

**FIGURE 1.**
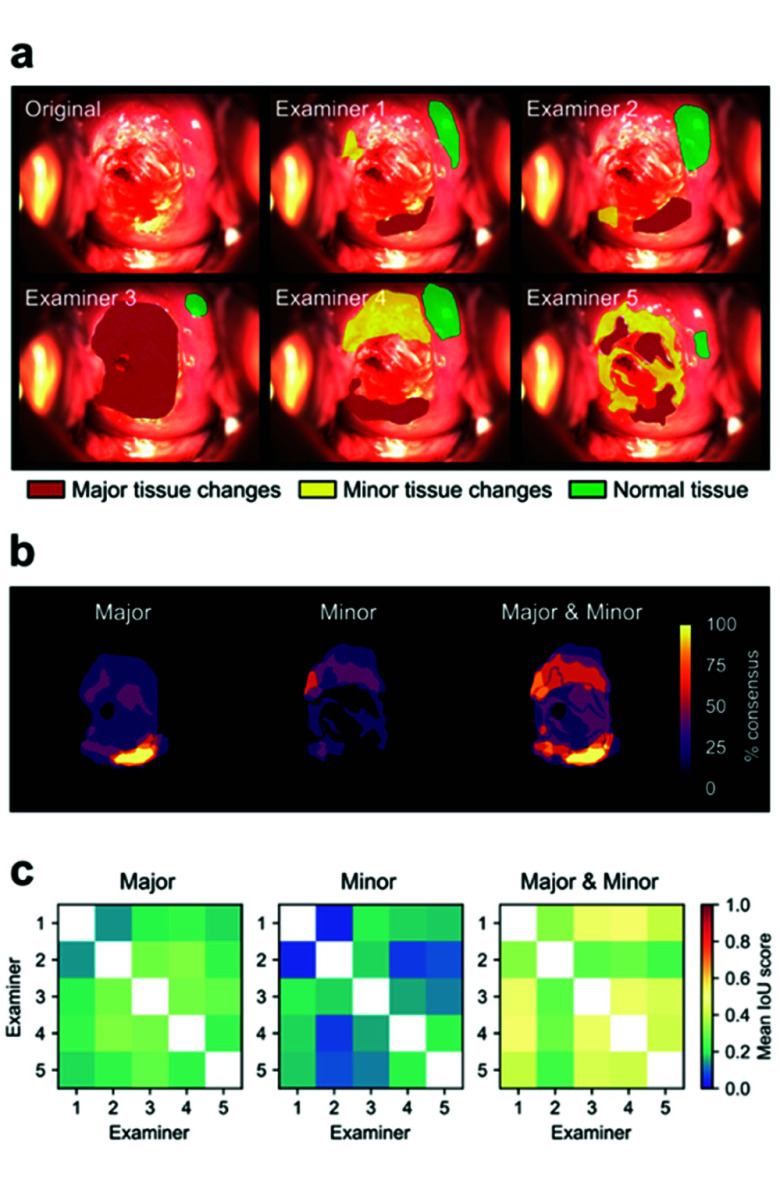
Annotations of CIN by medical experts. a) Example of a cervix image annotated by 5 examiners. Areas with major and minor tissue changes are marked with red and yellow respectively. A reference area without tissue changes is marked in green. b) Overlap of the annotations from a), with consensus between examiners expressed in percentages. c) Intersection over union (IoU) of annotations for pairs of examiners, averaged across patients (n = 11). Higher IoU scores correspond to higher annotation consensus among examiners.

Fig. 1a presents a representative cervix image alongside the annotations provided by all examiners. In this visualization, red and yellow denote areas with major and minor tissue changes, respectively, while green represents tissue without dysplastic alterations.

Fig. 1b illustrates the superimposed CIN annotations from Fig. 1a, categorized by the severity of tissue changes. The level of agreement among examiners is quantified as a percentage, where 100% indicates unanimous agreement on a tissue patch, while lower values reflect partial consensus. In this example, consensus was distributed over a broad range, with a small region reaching 100% agreement for major changes (Fig. 1b, left panel). In contrast, agreement for minor changes did not exceed 60% (Fig. 1b, middle panel). Overall, the broad distribution of consensus values underscores discrepancies among individual examiner annotations.

To objectively quantify the extent of annotation overlap, the IoU score was applied to pairwise annotations from different examiners. This metric ranges from 0 (no overlap) to 1 (perfect overlap). Fig. 1c depicts the distribution of IoU scores across examiner pairs, averaged across all patients for each category of tissue change. Values along the main diagonal are omitted, as they are always equal to 1. The mean IoU values indicate a low level of agreement among medical experts, particularly regarding the disease-degree-specific delineation of both major and minor tissue changes. Even when disregarding the degree of change — i.e., considering all CIN-affected areas collectively — the IoU values improve only marginally, reaching approximately 0.5. These findings highlight the substantial observer variability in colposcopic examination, even among experienced colposcopists, emphasizing the need for objective methodologies to enhance the consistency and reliability of CIN assessment.

### B. Hyperspectral Imaging Reveals Tissue Property Changes in CIN-Affected Areas

**FIGURE 2.**
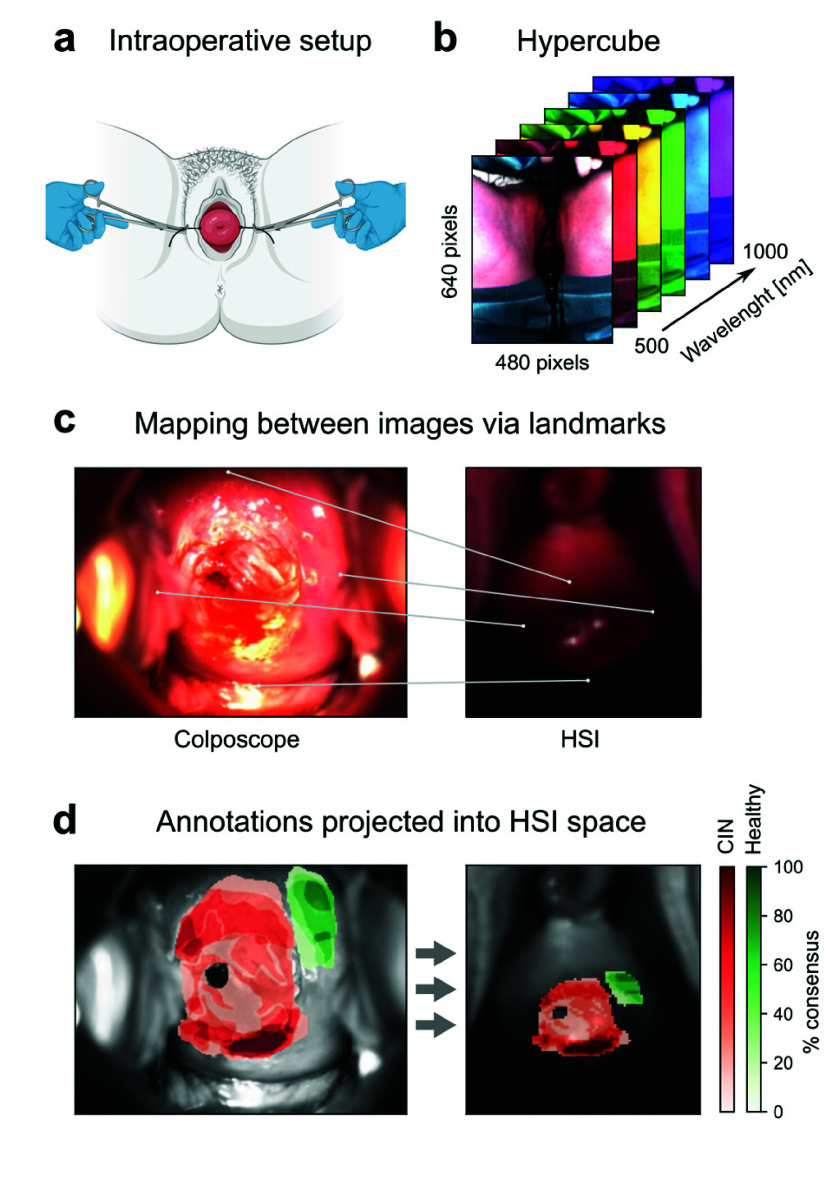
Hyperspectral imaging of the cervix. a) Schematic of intraoperative setup in preparation of hyperspectral imaging. b) HSI field of view and size of the measured hypercube. c) Left, high resolution image of the cervix acquired with a colposcope, right, zoomed-in section of the HSI image containing the cervix. Lines depict landmarks used to calculate a mapping between the two images. d) Left, annotation map for tissue with major and minor changes in red and no changes in green. Right, annotation map projected on the HSI image using the mapping between images calculated in c).

To evaluate whether HSI offers an improved method for identifying CIN-affected tissue, hyperspectral imaging of the cervix was performed using the TIVITA® Tissue System. Fig. 2a illustrates the intraoperative setup used to expose the cervix for HSI imaging. Each HSI measurement generates a set of images representing light reflectance at specific wavelengths, forming a three-dimensional data structure known as a hypercube. Fig. 2b provides an example of the HSI field of view and a schematic representation of the acquired hypercube.

By analyzing the subset of pixels corresponding to the cervix, it is possible to investigate molecular-level tissue properties that may indicate the extent of CIN involvement. To achieve this, the expert annotations had to be mapped onto the HSI image of the cervix. This was accomplished by establishing a spatial correspondence between the high-resolution colposcopic image, on which the annotations were made, and the associated HSI image. Landmarks visible in both images were used for alignment, as illustrated in Fig. 2c, where a high-resolution colposcopic image (left) is paired with the corresponding region of the HSI image (right). The alignment process, known as homography estimation, is a standard approach in computer vision. It determines a perspective transformation function based on landmark constraints, requiring a minimum of four corresponding landmarks per image. Once the transformation parameters were computed, any point from one image could be projected onto the other. This enabled the mapping of colposcopic annotations onto the HSI image. Fig. 2d shows an example of this projection, demonstrating that while the overall shape of the annotations was affected, their internal structures remained distinguishable, as highlighted by the dashed rectangles.

With the annotations transferred into the HSI space, a comparative analysis of hyperspectral properties was performed between CIN-affected and healthy tissue. To minimize variability caused by inter-observer discrepancies, annotations were thresholded to include only CIN-marked areas where at least 50% of the examiners reached consensus, ensuring that only regions consistently identified as CIN were considered.

**FIGURE 3.**
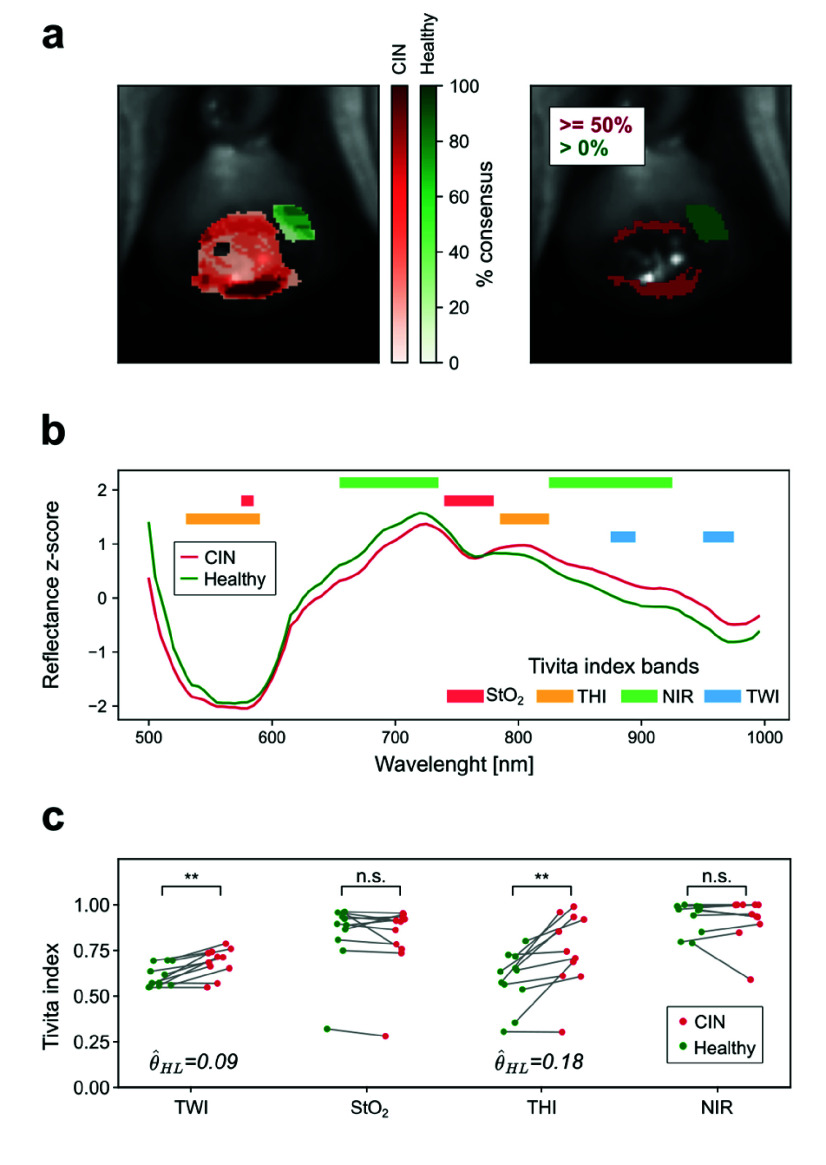
Spectral properties of CIN and healthy tissue. a) Left, examiners annotation map superimposed on the cervix from the HSI image. Right, areas with CIN tissue (red) where the consensus between examiners is high ( $> =50$%) and areas with healthy tissue (green). b) Tissue spectra averaged across the areas depicted in the right panel of a). Horizontal colored bars indicate the wavelength bands used by the TIVITA® Tissue System to calculate tissue properties such as oxygenation level (StO2), haemoglobin content (THI), perfusion (NIR) and water content (TWI). c) Changes in TIVITA indices between CIN and healthy tissue for all patients (n= 11). Gray lines connect measurement pairs from the same patient. $\Delta $ values show average index changes across all patients. $\ast \ast $ indicate statistical significance for the Wilcoxon signed-rank test. (TWI p= 0.005, THI p= 0.002). ${{\hat \theta }_{HL}}$ values show the Hodges–Lehmann estimator of the median difference (95% CI=[0.048, 0.095] for TWI and [0.046, 0.280] for THI).

For healthy tissue, the union of all marked areas was used, as these annotations were not exhaustive. Fig. 3a depicts the original annotation maps (left) and the thresholded maps (right) for a patient. The spectral data for the thresholded regions were then averaged. Fig. 3b displays the average spectra, standardized (z-scored) against their mean, for the example shown in Fig. 3a. Using the light absorption properties of certain biomolecules, the manufacturer of the HSI system defined a set of indices, known as the Tivita indices, to assess specific tissue properties. These include the TWI for water content, StO2 for oxygenation levels, THI for hemoglobin content, and NIR for tissue perfusion. Each index is calculated from predefined wavelength band ratios, as illustrated in Fig. 3b (horizontal colored bars).

The indices were computed for each pixel in the HSI image and averaged across the thresholded annotated regions (Fig. 3a right panel), for each patient. Fig. 3c shows the averaged Tivita indices paired by patient, for all patients (n= 11).

Statistical analysis using the Wilcoxon rank-sum test revealed significant differences in TWI (p= 0.005) and THI (p= 0.002) values between CIN-affected and healthy tissue. The Hodges–Lehmann estimator of the median difference showed that TWI indices typically increased by $\hat {\theta }_{HL}=0.09$ (95% CI=[0.048, 0.095]), while THI indices increased by $\hat {\theta }_{HL}=0.18$ (95% CI=[0.046, 0.28]), suggesting a more pronounced increase in THI compared with TWI. The standardized effect size was r= 0.80 for TWI and r= 0.86 for THI, indicating a large effect. These findings suggest that CIN-affected tissue contains significantly greater amounts of water and hemoglobin compared with healthy tissue.

The results remain consistent even when a higher consensus threshold of 75% was chosen, i.e. agreement among at least 4 of 5 examiners. The values for TWI were p= 0.003, $\hat {\theta }_{HL}=0.09$, 95% CI = [0.045, 0.114], r= 0.83; and for THI p= 0.001, $\hat {\theta }_{HL}=0.13$, 95% CI = [0.046, 0.318], r= 0.88. Taken together, the results provide promising preliminary evidence for the potential of HSI as a non-invasive and objective method for detecting CIN-affected tissue in clinical settings.

## IV. Discussions and Conclusion

Colposcopy remains the primary method for evaluating cervical abnormalities and plays a pivotal role in the screening, diagnosis, and management of CIN. Currently, the visualization of the cervical epithelium by colposcopy is performed by acetic acid and iodine staining which allows for the identification of tissue changes suggestive of neoplastic transformation. However, despite its widespread use, the interpretation of colposcopic findings is highly examiner-dependent, leading to substantial interobserver variability, even among experienced colposcopists. Although colposcopy is associated with high sensitivity in detecting abnormal cervical tissue, its specificity remains moderate, leading to frequent histological sampling to confirm diagnoses. The reliance on subjective visual assessment introduces variability that can impact patient outcomes, particularly in settings where access to skilled colposcopists is limited. Consequently, there is an urgent need for non-invasive, standardized approaches that can augment colposcopic evaluation, support in accurate clinical decisions and reduce unnecessary biopsies [20].

Various spectroscopic technologies have been explored in an effort to enhance the characterization of cervical tissue. For example, fluorescence and reflectance spectroscopy, have been investigated for their ability to detect molecular and structural tissue alterations associated with neoplasia [21], [22]. Some approaches focused on spectral changes following acetic acid application, leveraging its transient effects on light scattering properties to improve differentiation between normal and dysplastic tissue. While these advancements show promise, many rely on the application of contrast agents or chemical markers, which may introduce variability and limit their applicability across different anatomical sites [23]. Another technique that emerged as a powerful tool for non-invasive and marker-free tissue characterization is HSI. By capturing reflectance across a broad spectral range, HSI generates images that encode both spatial and spectral information about the underlying tissue. This technology has shown potential in various medical applications, including tissue differentiation, vascular assessment, and inflammation detection. Several research groups have investigated the utility of HSI in distinguishing normal from dysplastic cervical epithelium, yielding promising results [10], [24], [25], [26], [27]. Already in 2001 Ferris et al. reported reflectance measurements using an HSI probe on the ectocervix, demonstrating comparable specificity (70%) to the PAP smear but significantly higher sensitivity (97% vs. 72%). Mourant et al. identified three spectroscopic parameters in 29 patients that differentiated high-grade squamous intraepithelial lesions from low-grade lesions and normal tissue with 100% sensitivity and 80% specificity. Zheng et al. [27] analyzed hyperspectral data (600–800 nm) using a wide gap second derivative method and identified three specific wavelengths (620, 696, and 772 nm) for optimal tissue classification. They demonstrated the feasibility of classifying cervical tissue into normal, inflammation, and high-grade lesions, though the study was limited to only three patients.

Building on this previous research the current study aimed at identifying differences between CIN lesions and normal cervical tissue, with a particular focus on physiological tissue properties. Analysis of hyperspectral data within the 590 - 975 nm range, combined with medical expert tissue annotations, revealed significant increases in hemoglobin and water content for CIN lesions compared to normal tissue. The 50% examiner consensus was adopted as a pragmatic minimum level of agreement; repeating the analysis at a stricter 75% threshold produced consistent results, confirming that the observed differences are robust to variation of the consensus criterion. Angiogenesis is expected in the development and progression of neoplasms and may be related to malignant transformation into invasive cancer [28]. This could explain the increased levels in hemoglobin and water in CIN lesions.

These findings suggest that HSI may provide objectively quantifiable physiological markers for detecting cervical neoplasia, potentially improving diagnostic accuracy. Unlike opaque approaches such as deep learning, which still raise serious regulatory and interpretability limitations [29], [30], the use of physiological tissue properties enables more transparent clinical interpretation. Nonetheless, machine learning remains a powerful tool and could be harnessed by training algorithms on interpretable physiological features rather than raw hypercubes. For instance, tissue property values could be used to identify combinations that best correspond to a particular disease stage or subtype, while the spatial distribution tissue properties could be combined with convolutional neural networks to delineate the borders of diseased tissue. In this way, the pattern-recognition strength of these algorithms would be harnessed, while the results remain explainable and clinically meaningful. The findings of this study are consistent with recent advances in hyperspectral colposcopy reported by Vega et al., who demonstrated the feasibility of an integrated hyperspectral colposcope for detecting cervical lesions in a cohort of 62 patients [31]. Their system captured 158 spectral bands across the 470-900 nm range and revealed statistically significant spectral differences between exocervix, endocervix, and cancerous tissue, particularly in wavelength regions associated with hemoglobin absorption. These observations align with the increased hemoglobin and water content detected in CIN lesions in the present study, reinforcing the potential of HSI to identify physiological changes associated with neoplastic transformation. While their approach utilized unsupervised segmentation via principal component analysis, the present study employed direct physiological parameter extraction, potentially offering greater clinical interpretability. Both methodologies underscore HSI’s capacity to provide objective, quantifiable biomarkers that could reduce examiner-dependent variability and support the development of standardized diagnostic protocols for cervical cancer screening.

While the results of this study are encouraging, several limitations must be acknowledged that impact the immediate clinical applicability of the findings. First, the small patient cohort (n= 11) restricts the generalizability of the findings and precludes the establishment of robust diagnostic thresholds. Large-scale prospective trials incorporating diverse patient populations are necessary to validate these results. Second, CIN labelling relied solely on visual identification by the medical experts. As this study shows, consensus among experts is moderate at best, therefore not ideal for defining ground truth. The lack of histopathological confirmation for all identified regions represents a weakness, as visual assessment – even by experts – cannot definitively distinguish between CIN grades or exclude benign mimics. A better approach would be to confirm the visual tissue identification using more objective CIN diagnostic methods, such as through colposcopically-directed biopsies with subsequent histopathological correlation. Third, the use of two different imaging systems and the subsequent mathematical transformation between them introduce variability, potentially affecting the accuracy of the results. This approach was necessary due to the lack of a HSI system optimized for close-up imaging. The TIVITA® system used in this study, originally developed for perfusion and wound assessment, had a fixed tissue-camera distance of approximately 50 cm, leading to a reduced effective imaging area and lower-resolution HSI images of the cervix. Additionally, intraoperative imaging required manual cervical stabilization, limiting the direct applicability of this system in routine colposcopic examinations. While future development of a dedicated endoscopic HIS system appears technically feasible, significant engineering challenges remain, including real-time data processing, and integration into existing colposcopic workflows without disrupting clinical practice. This proof-of-principle study does not aim to present a solution ready for immediate integration into standard colposcopic examinations but rather to demonstrate that such a solution may be attainable with further development. Future work should focus on developing an endoscopic HSI system with a shorter working distance capable of real-time spectral analysis and high spatial resolution, as well as on validating the current findings in larger and more diverse populations to establish robust thresholds for separating CIN-affected from healthy tissue. Lastly, due to the limited amount of data and the inherent variability of measurements, the analysis was restricted to mean spectra comparisons, which may overestimate statistical test performance. This analytical approach, while necessary given the dataset size, cannot capture the full complexity of tissue heterogeneity and may obscure clinically relevant intra-lesion variability. A larger, finer grained dataset would enable more complex analyses, capable of capturing better the variability and spatial distribution of the data and ultimately enhancing diagnostic accuracy.

In conclusion, this study demonstrates the potential of HSI as a non-invasive tool for CIN assessment, able to enhance the accuracy of colposcopic diagnosis. Ongoing research is directed toward addressing the hardware limitations of currently available HSI systems and optimizing their integration into routine gynecological practice. With these advancements, HSI could become a valuable addition to cervical cancer screening, improving diagnostic consistency and ultimately enhancing patient care in gynecological oncology.
